# Inverted Social Reward: Associations between Psychopathic Traits and Self-Report and Experimental Measures of Social Reward

**DOI:** 10.1371/journal.pone.0106000

**Published:** 2014-08-27

**Authors:** Lucy Foulkes, Eamon J. McCrory, Craig S. Neumann, Essi Viding

**Affiliations:** 1 Department of Clinical, Educational and Health Psychology, University College London, London, United Kingdom; 2 Department of Psychology, University of North Texas, Denton, Texas, United States of America; Nathan Kline Institute and New York University School of Medicine, United States of America

## Abstract

Individuals with high levels of psychopathic traits tend to undervalue long-term, affiliative relationships, but it remains unclear what motivates them to engage in social interactions at all. Their experience of social reward may provide an important clue. In Study 1 of this paper, a large sample of participants (N = 505) completed a measure of psychopathic traits (Self-Report Psychopathy Scale Short-Form) and a measure of social reward value (Social Reward Questionnaire) to explore what aspects of social reward are associated with psychopathic traits. In Study 2 (N = 110), the same measures were administered to a new group of participants along with two experimental tasks investigating monetary and social reward value. Psychopathic traits were found to be positively correlated with the enjoyment of callous treatment of others and negatively associated with the enjoyment of positive social interactions. This indicates a pattern of ‘inverted’ social reward in which being cruel is enjoyable and being kind is not. Interpersonal psychopathic traits were also positively associated with the difference between mean reaction times (RTs) in the monetary and social experimental reward tasks; individuals with high levels of these traits responded comparatively faster to social than monetary reward. We speculate that this may be because social approval/admiration has particular value for these individuals, who have a tendency to use and manipulate others. Together, these studies provide evidence that the self-serving and cruel social behaviour seen in psychopathy may in part be explained by what these individuals find rewarding.

## Introduction

Psychopathy is a personality disorder characterised by lack of empathy, shallow affect and callous treatment of other people, as well as impulsivity and a greater propensity towards criminal behaviour [1]. Psychopathic traits are continuously distributed in the population and can be reliably measured in community samples [2]–[3].

Empirical evidence suggests that psychopathic traits may be associated with an atypical experience of social reward [4]–[9]. Social reward can be defined as the motivational and pleasurable aspects of our interactions with other people, and interpersonal kindness and closeness is a fundamental social reward for most people [10]–[11]. However, it appears that individuals with high levels of psychopathic traits do not place equal importance on affiliative, long-term friendships and relationships [4]. Instead they favour friends who can increase their access to sexual mates or provide protection [12] and prefer one-night stands to committed relationships [13]. In addition, evidence from experimental tasks shows that individuals with high levels of psychopathic traits are less likely to cooperate with and help others [5], [8]–[9] Together, this evidence suggests that, for individuals with high levels of psychopathic traits, affiliative and prosocial behaviour towards others may be less rewarding than it is for typical individuals [6].

Furthermore, psychopathic traits are associated with enjoyment of antisocial entertainment such as violent sports and video games [14] and internet ‘trolling’ - online antisocial behaviour [15]. This evidence suggests individuals with high levels of psychopathic traits not only lack empathy towards others’ distress [16]–[17], but may actually take pleasure from it. Thus individuals with high levels of psychopathic traits appear to show an unusual pattern of social reward: decreased reward value of prosocial and affiliative interactions [4], [6]–[7], and increased reward value of cruelty towards others [14]–[15]. This suggestion is supported by a recent study that used a systematic measure of social reward, the Social Reward Questionnaire (SRQ; [7]). This preliminary analysis found that scores on the psychopathy subscale of the brief Dirty Dozen measure [18] were negatively associated with enjoyment of prosocial interactions and positively associated with enjoyment of callous, antisocial interactions [7]. However, this four-item measure of psychopathy is unidimensional, so it remains unclear how different aspects of psychopathic personality are associated with dimensions of social reward. As such, there remains a need to systematically explore associations between the value of different social rewards and a comprehensive, well-validated measure of psychopathic traits.

There is an equal need to employ experimental measures that can more sensitively assess the experience of social reward in relation to psychopathic traits. Such measures have the potential to overcome several of the limitations inherent in using self-report questionnaires, including the ability and/or willingness of participants to reflect on and state their personality traits. Some research has assessed responsiveness to *monetary* reward in relation to psychopathic traits, and found that individuals with high levels of these traits may be hyperresponsive to this type of reward [19]–[20]. Although the last decade has seen a surge in the number of studies using experimental paradigms to measure social reward (e.g. [21]–[23]), to our knowledge these paradigms have not yet been used in association with a measure of psychopathic traits.

In the current paper, we report two studies that explore the relationship between social reward and psychopathic traits. In the first study, we aimed to assess the association between subtypes of social reward and dimensions of psychopathic traits using questionnaire methodology. In the second study, our aim was to employ an experimental measure of social reward and investigate its association with psychopathic traits (Study 2).

## Study 1

In Study 1, our aim was to elucidate some of the processes that may motivate the unpleasant interpersonal behaviour associated with psychopathic traits. To do this, we explored associations between psychopathic traits, as measured by the Self-Report Psychopathy Scale Short-Form (SRP-SF; [24]), and the value of different types of social reward, as measured by the Social Reward Questionnaire (SRQ; [7]). The SRP-SF measures four dimensions of psychopathy: Affective (e.g. lack of empathy), Interpersonal (e.g. manipulativeness), Lifestyle (e.g. impulsivity) and Antisocial (e.g. aggressive or unlawful behaviour). The SRQ quantifies the enjoyment of six types of social reward: Admiration (being flattered and gaining attention), Negative Social Potency (being cruel and callous), Passivity (allowing others control), Prosocial Interactions (being kind and fair), Sexual Relationships (frequent sexual encounters) and Sociability (frequent socialising). We hypothesised that psychopathic traits would be positively associated with Negative Social Potency and negatively associated with Prosocial Interactions. In addition, we hypothesised that psychopathic traits would be positively associated with Sexual Relationships, due to the high rates of affairs and short-term relationships reported in this group [25]–[26]. Finally, we predicted that psychopathic traits would be positively associated with enjoyment of Admiration, due to the elevated levels of narcissism seen in individuals with high levels of psychopathic traits [27]. We made no specific hypotheses regarding which dimensions of psychopathy would show these associations. Associations between psychopathic traits and other types of social reward were exploratory.

### Materials and Methods

Data for this study were collected as part of a wider battery of measures that have been partly reported in a previous publication [7].

#### Ethics Statement

All participants provided written informed consent and the study was approved by the University College London Clinical, Educational and Health Psychology Research Ethics committee.

#### Participants

Amazon’s Mechanical Turk platform (MTurk) was used to recruit participants. MTurk is an international crowdsourcing website on which participants complete tasks or questionnaires for payment, and is increasingly being used as a source of valid and reliable data [28].

The questionnaires were completed 529 times. Participants were excluded for providing obviously repetitive answers (i.e. giving the same answer to all questions in at least three of the six questionnaires in the original battery; N = 5) or for completing the questionnaire battery twice (second attempt excluded; N = 19). This left a final sample of 505 participants (270 males, 235 females) aged 18 to 79 years (mean = 34.0, SD = 12.2). The majority of respondents lived in the USA (N = 457); other respondents lived in India (N = 35), Canada (N = 6), the UK (N = 6) or another European country (N = 1). The ethnicity of the sample was as follows: 72.3% White, 11.1% South Asian, 6.1% Black, 2.8% Hispanic, 2.0% East Asian and 5.7% Mixed/Other. The highest completed education level of the sample was as follows: 38.2% Bachelor’s degree, 30.9% Senior/high school, 18.8% College, 12.1% Postgraduate degree.

#### Measures

Psychopathic traits: these were measured with the Self-Report Psychopathy Scale Short Form (SRP-SF; [24]), a well-validated instrument modelled on the Psychopathy Checklist Revised (PCL-R; [1]). The SRP-SF contains 28 items that participants rate on a 5-point Likert scale (1 = Strongly disagree to 5 = Strongly agree). The SRP-SF yields scores for four dimensions of psychopathy: Affective (e.g. lack of empathy), Interpersonal (e.g. manipulativeness), Lifestyle (e.g. impulsive) and Antisocial (e.g. harmful and potentially criminal behaviour). There are seven items for each of the four dimensions, which can be summed to form a total psychopathy score. We chose to use the SRP-SF rather than the original SRP as it takes less time to complete, whilst still retaining strong psychometric properties [24].

The SRP-SF and the SRP on which it is based both have good basic psychometrics, as well as theoretically sound and mathematically strong latent structures [2], [6], [17], [29]–[33]. There is good evidence for convergent validity between the SRP/SRP-SF and other measures of psychopathic traits. For example, both measures are strongly positively correlated with the PCL-R and also have the same four-factor structure [24], and three factors of the SRP-SF (Interpersonal, Affective, Lifestyle) are strongly correlated with the three factors of the Youth Psychopathic Traits Inventory (Grandiose/Manipulative, Callous/Unemotional, Impulsive/Irresponsible; [32]). Finally, SRP subscales are strongly correlated with expected subscales of the Elemental Psychopathy Assessment, a measure of psychopathic traits based on the five-factor model of personality (EPA; e.g. SRP Interpersonal is strongly correlated with EPA Manipulation and Self-Centeredness [30]).

Across a wide diversity of samples, the SRP traits are associated in the expected theoretical directions with relevant external correlates, such as criminal offenses and externalizing psychopathology [32], [34]–[37], moral reasoning [17], amygdala activation to fearful faces [29], and lower amygdala volume [38]. The construct validity of both the SRP and SRP-SF are further supported by studies demonstrating theoretically meaningful associations with related personality measures [31], [33], as well as cognitive functioning [2], social information processing [16], and social functioning [6]. Based on the use of a mega world-sample (30 k+), latent variable model-based research with the SRP has shown it to be invariant across sex, and the SRP factors were associated with world regional data such as Gross Domestic Product (GDP), fertility, and infant mortality [39]. In the current sample, Cronbach’s Alpha scores indicated acceptable to good reliability (mean = .76, SD = .10; Affective = .76, Interpersonal = .86, Lifestyle = .80, Antisocial = .61).

Social reward: the Social Reward Questionnaire (SRQ; [7]) is a 23-item scale used to measure individual differences in the value of social rewards. Each item begins “I enjoy” and then describes a different type of social interaction. Participants are asked to consider the item in relation to all their social interactions, e.g. friends, partners, family, colleagues or people they have just met. Responses are given on a 1 to 7 scale (1 = Disagree strongly, 7 = Agree strongly). The SRQ consists of six subscales, each representing a domain of social reward: Admiration, Negative Social Potency, Passivity, Prosocial Interactions, Sexual Relationships and Sociability (see Table 1). In the current sample, Cronbach’s Alpha scores indicated good reliability (mean = .82, SD = .04; Admiration = .82, Negative Social Potency = .87, Passivity = .78, Prosocial = .84, Sexual = .84, Sociability = .77).

**Table 1:** Detail of SRQ subscales.

SRQ subscale	Description	Example item
***Admiration***	Being flattered, liked and gaining positive attention	*“I enjoy achieving recognition from others”*
***Negative Social Potency***	Being cruel, callous and using others for personal gains	*“I enjoy embarrassing others”*
***Passivity***	Giving others control and allowing them to make decisions	*“I enjoy following someone else’s rules”*
***Prosocial Interactions***	Having kind, reciprocal relationships	*“I enjoy treating others fairly”*
***Sexual Relationships***	Having frequent sexual experiences	*“I enjoy having an active sex life”*
***Sociability***	Engaging in group interactions	*“I enjoy going to parties”*

#### Data analysis procedure

Pearson and Spearman correlational analyses (as appropriate depending on the normality of the bivariate residuals) were conducted using IBM SPSS Statistics 20.0 for Windows. Scores for the four psychopathy factors and the total psychopathy score were correlated with all SRQ subscales using zero-order correlations. Benjamini and Hochberg False Discovery Rate [40] was used to control for the probability of making a Type I error on multiple comparisons, and only corrected p-values are presented. There were no missing data, as the questionnaire was programmed in such a way that all items required a response.

### Results

Descriptives for SRQ and SRP-SF scores are shown in Table S1. Results from the correlational analyses are shown in Table 2. All psychopathy scores were positively associated with the Negative Social Potency subscale of the SRQ and negatively associated with the Prosocial Interactions subscale. All psychopathy scores except the Antisocial factor were positively associated with Sexual Relationships, and all except the Affective factor were positively associated with Passivity. Finally, Lifestyle psychopathic traits were positively associated with Sociability, and Interpersonal psychopathic traits were positively associated with Admiration.

**Table 2:** Correlations between SRP and SRQ scores in Study 1 (N = 505).

	SRP subscale				SRP Total^a^
	Affective^a^	Interpersonal^a^	Lifestyle^a^	Antisocial^b^	
*SRQ subscale*					
Admiration	.01	.10*	.07	−.06	.05
Negative Social Potency	.63**	.65**	.50**	.60**	.70**
Passivity	.08	.12*	.11*	.13**	.14**
Prosocial Interactions	−.43**	−.39**	−.27**	−.45**	−.45**
Sexual Relationships	.15**	.14**	.34**	.05	.20**
Sociability	.00	.07	.15**	.07	.08

a Zero order Pearson correlations are reported.

b Zero order Spearman correlations are reported.

Corrected p values are shown.

*p<.05,

**p<.01.

#### Post-hoc analyses

Previous evidence has shown that age and gender can affect both reward processing (age: [41]; gender: [42]) and level of psychopathic traits (age: [43]; gender: [44]). We therefore conducted post-hoc analyses to explore possible effects of age and gender on the associations between psychopathic traits and social reward (see Tables S5–S7).

#### Age

We re-ran the correlations between the two measures as partial correlations, controlling for age (see Table S5). When age is controlled, the following associations are no longer significant: Admiration and Interpersonal psychopathic traits (r = .06, adjusted p = .24) and Passivity and Lifestyle psychopathic traits (r = .09, adjusted p = .07), and the association between Admiration and Antisocial psychopathic traits becomes significant (r = −.10, adjusted p<.05). However, the pattern of associations largely remained the same.

#### Gender

We re-ran the correlations between the two measures in Study 1 for each gender independently. We then used the Fisher r-to-z transformation to assess if the differences between associations for each gender were significant (see Tables S6 and S7). The pattern of associations was largely the same for males and females, but the differences are worthy of note. Firstly, females showed a stronger association between Sexual Relationships and Affective psychopathic traits (z = 2.19, p<.05). Four associations were significantly stronger in males than females: Passivity and Antisocial psychopathic traits (z = 2.79, p<.01), Sexual Relationships and Antisocial psychopathic traits (z = −2.86, p<.01), SRQ Sociability and Interpersonal psychopathic traits (z = 2.14, p<.05) and Sociability and Total psychopathic traits score (z = 2.05, p<.05).

### Study 1 Discussion

All psychopathic traits were positively associated with Negative Social Potency and negatively associated with Prosocial Interactions. This supports our hypothesis of an ‘inverted’ pattern of social reward in individuals with high levels of psychopathic traits, in which being cruel is enjoyable and being kind is not. Affective, Interpersonal and Lifestyle psychopathic traits were positively associated with enjoyment of Sexual Relationships, consistent with our hypothesis and in line with previous evidence of increased promiscuity in these individuals [25]–[26]. In addition, there was a positive association between Interpersonal psychopathic traits and enjoyment of Admiration. The Interpersonal psychopathy factor includes manipulativeness and superficial charm, and we speculate that an admiring individual would be more susceptible to this manipulative control. Therefore, gaining others’ admiration could facilitate the self-serving social strategy of individuals with high levels of Interpersonal psychopathic traits, instilling this social interaction with high reward value. Additionally, admiration may be rewarding because it feeds the narcissistic traits associated with Interpersonal psychopathic traits [45].

There were positive associations between Interpersonal, Lifestyle and Antisocial psychopathic traits and enjoyment of Passivity. We speculate this may be due to the parasitic relationship style of individuals with high levels of psychopathic traits [1], [12], which may lead these individuals to enjoy social interactions in which another person expends effort to bring them gains. Lastly, there was a positive association between Lifestyle psychopathic traits and Sociability. We speculate that individuals with high levels of Lifestyle psychopathic traits may enjoy socialising with others because this provides a context for the risk-taking and sensation-seeking behaviours that this factor represents [1]. For example, attending parties may increase the opportunity to take recreational drugs.

Our post-hoc analyses revealed some interesting effects of age and gender, although the pattern of associations between social reward and psychopathic traits largely remained the same. Overall, the associations found here between dimensions of psychopathic traits and different types of social reward provide evidence for possible motivations behind the patterns of social behaviour seen in psychopathy.

## Study 2

In Study 2, we tested a sample of UK participants in person. The first goal of this study was to explore the associations that we found between social reward and psychopathic traits in Study 1 in a different sample. The second goal was to use two experimental reward tasks to assess how monetary and social reward value relates to psychopathic traits. These experimental tasks were intended to provide a sensitive index of reward value that would be less susceptible to possible impression management than could be the case for self-report measures such as the SRQ. The tasks also allowed social reward to be explored in the context of another type of salient reward, money.

Tasks that compare responses to monetary and social reward are already available (e.g. [41]–[42], [46]). However, the stimuli used to represent the two types of reward are conceptually and perceptually different from each other, which somewhat complicates the interpretation of the findings from these studies. For example, one study [46] represented monetary reward often with a currency symbol (a dollar sign), a simple conceptual representation for which an association with reward has been learned over time. In contrast, social reward was represented with a smiling face: a visually complex, biologically salient image [46]. In order to comparably address individuals’ relative processing of monetary and social reward, there is a need to use stimuli that allow these two rewards to be represented as equally as possible. To address this issue in the current study, social reward was represented using the ‘Like’ symbol from the social networking site Facebook (www.facebook.com). This is a thumbs-up symbol used to express approval/admiration from one user to another in response to user-posted items, such as photos or comments. We then used a pound sterling symbol to represent monetary reward, and using these symbols together has two benefits. Firstly, both the Like and pound symbols are images that have a learnt association with reward. In other words, these symbols both indicate a conceptual representation of reward. Secondly, both symbols have similar, abstract visual features. Together, these characteristics allow us to compare the relative processing of monetary and social reward value as validly as possible.

Existing studies of monetary reward value have shown that psychopathic traits are positively associated with increased activity in reward-related brain areas, such as the nucleus accumbens, when processing monetary reward [19]–[20]. In addition, behavioural research has found positive associations between psychopathic traits and importance of life goals relating to money [6]. We therefore hypothesised that psychopathic traits would be positively associated with reaction times (RTs) to reward in the monetary task. With regard to social reward, findings from Study 1 of this paper suggest that psychopathic traits are associated with less reward from prosocial interactions. On the basis of this, we hypothesised that psychopathic traits would be negatively associated with RTs to reward in the social task. Finally, we hypothesised that psychopathic traits would be negatively associated with a monetary–social RT difference score (i.e. RTs to social reward will be relatively slower than those to monetary reward). Based on the findings from Study 1, we hypothesised that all psychopathy factors would show this pattern of association.

### Materials and Methods

#### Ethics Statement

All participants provided written informed consent and the study was approved by the University College London Clinical, Educational and Health Psychology Research Ethics committee.

#### Participants

Participants were 110 males recruited from two participant pools at University College London (UCL): the UCL Psychology Subject Pool and the ICN (Institute of Cognitive Neuroscience) Subject Database. Both pools are open to students across the university and to members of the public. Only males were recruited due to the higher prevalence of psychopathic traits in males and to ensure we did not lose power in the relatively small sample size by controlling for another variable (gender). Participants were aged 18–39 years (mean = 22.45, SD = 4.07) and all met the following criteria: fluent English-speaker, no dyslexia and a current Facebook user. Ninety percent of the sample were current students (6.4% unemployed, 3.6% employed) and all lived in the UK. The highest completed education level was as follows: 65.4% senior school/A level college, 19.1% Bachelor’s degree, 15.5% postgraduate degree. Ethnicity of the sample was as follows: 28.2% Chinese, 21.8% White other, 20.9% Mixed/Other, 19.1% White British, 10.0% Indian.

#### Questionnaires

Psychopathic traits: the SRP-SF [24] was used to measure psychopathic traits as in Study 1.

Social reward: the SRQ [7] was used to measure the value of different types of social reward as in Study 1.

Facebook usage: use of the social media website Facebook was measured with the Facebook Intensity Scale [47]. This is an 8-item questionnaire that assesses frequency and duration of Facebook usage as well as emotional connectedness to the site. This measure was given in order to control for the effect of Facebook usage on the reward value of the ‘Like’ symbol in the experimental social reward task.

#### Monetary and social reward tasks

Two versions of a probabilistic reward anticipation task (monetary and social) were used. These tasks were based on the Factorial Reward Anticipation task [48] and the Monetary Incentive Delay task [49]. The monetary and social tasks were conducted separately (rather than as part of one task) for two reasons. Firstly, separating the two tasks with a battery of questionnaires in-between reduced the possibility of boredom or fatigue effects. Secondly, conducting separate tasks removed the effect of shifting costs that could incur if participants had to change frequently between the two symbolic representations. Comparing two types of reward by using two separate tasks has been done previously (e.g. [21]).

**Figure 1:**
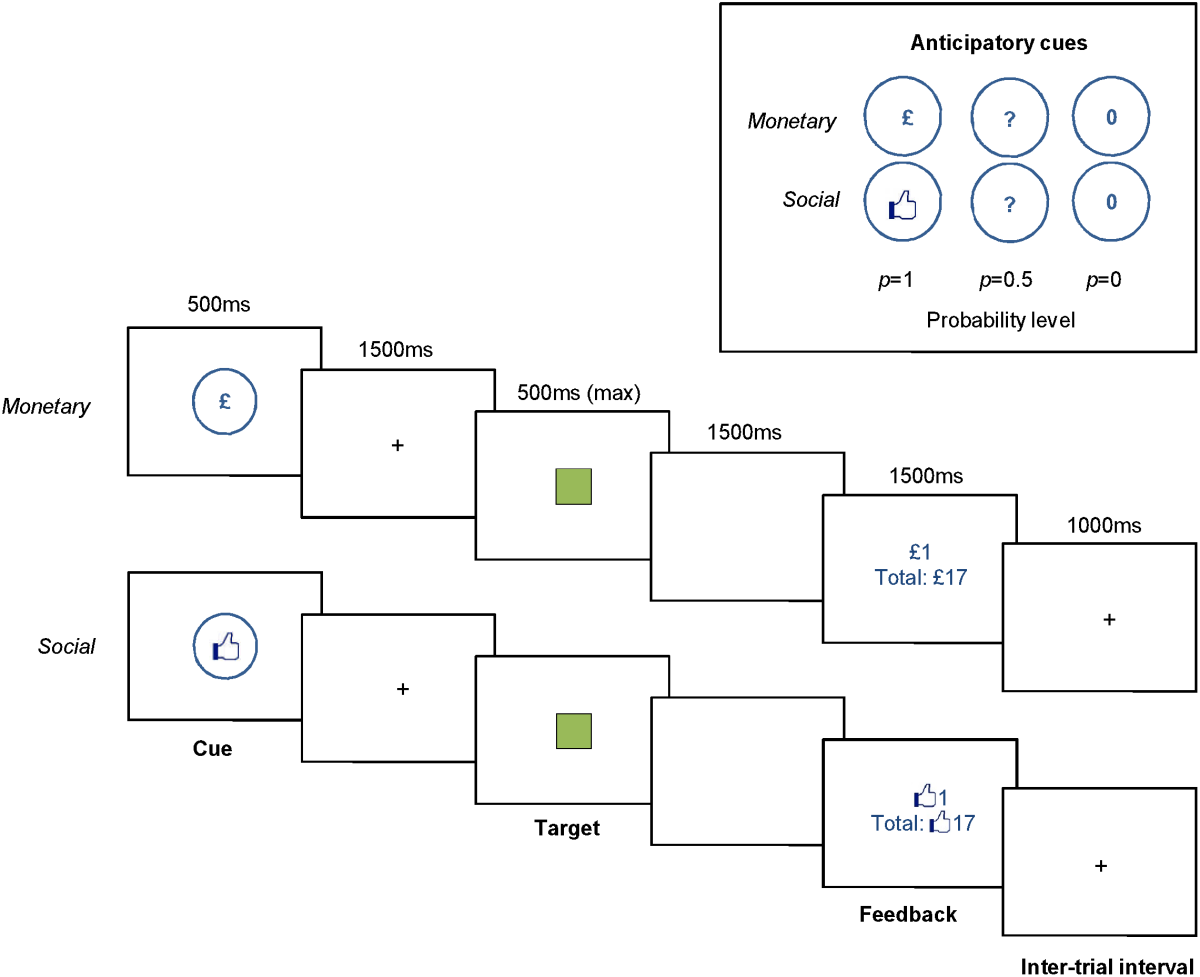
Monetary and social reward task trial sequences.

In both tasks, a cue indicates how likely it is that a key press response will yield rewarding feedback. The participant then responds to a target by pressing the space bar, and subsequently receives feedback, which is either reward (a monetary or social point gain) or no reward (no point gain; there is no loss condition). Therefore, each trial has 6 sequential components: (1) 500 ms anticipatory cue, (2) 1500 ms fixation cross, (3) 500 ms green square target, (4) 1500 ms blank screen, (5) 1500 ms feedback, (6) 1000 ms inter-trial interval (each trial is 6.5 seconds). There are three possible cues, shown in Figure 1, which indicate to the participant that there is a p = 0, p = 0.5 or p = 1 probability level of receiving a point in that trial, provided they press the space bar quickly (within 500 ms) when the target appears. If the space bar is pressed within 500 ms on a rewarded trial (i.e. in 100% of the 1 probability trials and a randomised 50% of the 0.5 probability trials), ‘+1′ is presented with the reward symbol (either a pound or Like symbol). If the space bar is not pressed, is pressed outside of the 500 ms window, or is pressed within the 500 ms window but on a no-reward trial (i.e. in all 0 probability trials and 50% of 0.5 probability trials), ‘+0′ is presented with the reward symbol. On each feedback screen, cumulative winnings are shown underneath the trial winnings (see Figure 1). Within each task, the sequence of trials (0, 0.5 or 1) was randomised for each participant.

It is worth noting that no actual reward was awarded on the basis of task performance. Participants were given a flat rate of £10 for taking part in the study, and were told that the objective of the reward tasks was simply to earn as many points as possible. We made this decision because we wanted to keep the two tasks as equivalent as possible (i.e., translating the monetary points into winnings in the monetary condition could not be matched in the social condition). Therefore, we relied on the learned association between the two symbols (pound sign and Like symbol) and reward value. This is in line with other studies comparing the two types of reward, where winnings are not translated into actual monetary reward [41], [50].

#### Procedure

Participants completed the questionnaires and monetary and social reward tasks as part of a wider data collection. One experimental reward task (either money or social; counterbalanced across participants) appeared at the beginning of the battery and the other appeared at the end (approximately 40 minutes apart).

#### Data analysis procedure

Zero order correlational analyses were used to assess associations between SRP-SF and SRQ, as in Study 1. Benjamini and Hochberg False Discovery Rate [40] was used to control for the probability of making a Type I error on multiple comparisons, and only corrected p-values are presented. There were no missing data, as the questionnaire was programmed in such a way that all items required a response.

In the experimental reward tasks, trials with RTs that were <100 ms or >1000 ms (including any missing trials) were excluded from analysis. According to these criteria, eight participants had >20% invalid trials in either the monetary or social reward task and were excluded from analysis, giving a final sample size of N = 102.

Mean reaction times (RTs) for each probability level (0, 0.5 and 1) were calculated in both conditions (monetary and social) for each participant. In addition, a difference score was calculated that represented the relative value of the monetary and social conditions. To do this, the mean score for each probability level in the social condition was deducted from the corresponding mean score in the monetary condition.

We first compared general task performance on the monetary and social tasks. A 2 (reward type: monetary, social)×3 (reward probability: 0, 0.5, 1) ANOVA was conducted to investigate this. To explore associations between psychopathic traits and performance on the experimental reward tasks, correlational analyses were run between the psychopathy factor and total scores and the mean RTs and monetary-social difference scores from the experimental tasks. Benjamini and Hochberg False Discovery Rate [40] was used, and only corrected p-values are presented.

### Results

#### Questionnaires

Descriptives for SRQ and SRP-SF scores are shown in Table S2. The four psychopathy factor scores (Affective, Interpersonal, Lifestyle and Antisocial) and total psychopathy score were all positively associated with Negative Social Potency, as in Study 1 (see Table 3). Affective and Antisocial factors were negatively associated with Prosocial Interactions. All scores except the Antisocial factor were positively associated with Sexual Relationships. Finally, only the Interpersonal factor was positively associated with Passivity and Admiration, and there were no significant associations with Sociability.

**Table 3:** Correlations between SRP and SRQ scores in Study 2 (N = 110).

	SRP-SF subscale				SRP-SF Total^a^
	Affective^a^	Interpersonal^a^	Lifestyle^a^	Antisocial^b^	
*SRQ subscale*					
Admiration	.06	.21*	.10	−.09	.12
Negative Social Potency	.56**	.60**	.36**	.32**	.58**
Passivity	.18	.20*	.07	−.03	.15
Prosocial Interactions	−.26*	−.02	−.12	−.22	−.19
Sexual Relationships	.30**	.31**	.45**	.16	.41*
Sociability	−.05	.05	.21	.00	.08

a Zero order Pearson correlations are reported.

b Zero order Spearman correlations are reported.

Corrected p values are shown.

*p<.05,

**p<.01.

#### Monetary and social reward tasks

**Figure 2:**
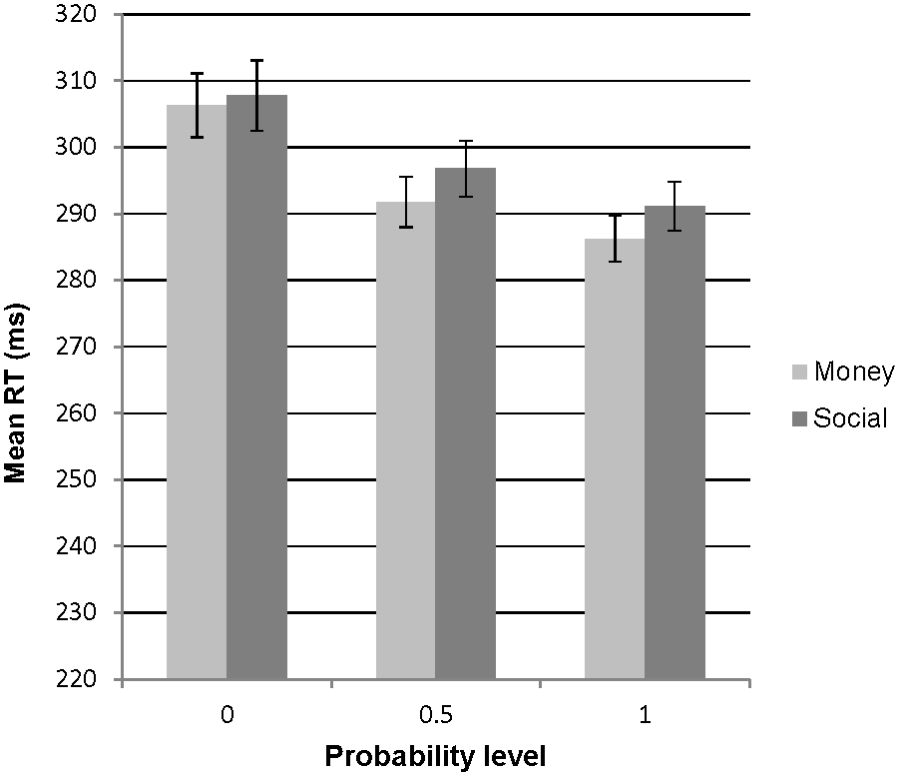
Plot of mean RTs for each probability level in both monetary and social conditions. N.B. Error bars represent standard error.

Descriptives of RTs for each probability level in monetary and social tasks can be found in Table S3. Mean RTs were analysed with a 2 (reward type: monetary, social) ×3 (reward probability: 0, 0.5, 1) ANOVA. There was a significant main effect of reward probability (F(1,101) = 38.82, p<.001; see Figure 2); participants responded more quickly to increased probability of reward. Analysis of simple effects showed that the decrease in RT between increases in reward probability (0 and 0.5; 0.5 and 1) were both significant, in both monetary and social conditions (all p<.05; see Table S4). There was no main effect of reward type and no interaction between reward type and reward probability.

#### Associations between psychopathic traits and performance on reward tasks

Degree of Facebook usage as measured by the Facebook Intensity Scale [44] was entered as a control variable in all analyses, and Benjamini and Hochberg False Discovery Rate [40] was used to control for the probability of making a Type I error on multiple comparisons.

There were no significant associations between psychopathy scores and mean RTs at any probability level in either the monetary or social task. However, Interpersonal psychopathic traits were significantly positively associated with the RT *difference* scores for the 0.5 and 1 probability conditions. Specifically, as Interpersonal traits increased, RTs to the social condition were faster relative to the monetary condition (see Table 4).

**Table 4:** Correlations between SRP scores and reward task RTs and difference scores.

		SRP-SF subscale				SRP-SF Total^a^
		Affective^a^	Interpersonal^a^	Lifestyle^a^	Antisocial^b^	
	*Probability*					
*Monetary*	0	.08	−.02	−.03	.03	−.01
	0.5	.03	.02	.00	−.05	.00
	1	.04	.09	−.01	−.11	.01
*Social*	0	.03	−.11	−.15	−.07	−.14
	0.5	.01	−.20	−.15	−.12	−.16
	1	.05	−.14	−.15	−.09	.13
*Monetary-Social* c	0	.08	.13	.18	.14	.18
	0.5	.02	.30*	.22	.12	.23
	1	−.02	.27*	.17	.00	−.16

a Pearson correlations are reported.

b Spearman correlations are reported.

c Difference score calculated by subtracting mean RT in social condition from mean RT in monetary condition.

Facebook usage controlled for in all analyses. Corrected p values are shown.

*p<.05.

### Study 2 Discussion

In Study 2, the pattern of associations between psychopathic traits and social reward found in Study 1 was largely replicated. Specifically, in both samples there was a positive association between all psychopathy scores and Negative Social Potency, the enjoyment of being cruel and controlling towards others. Both studies found positive associations between Affective, Interpersonal, Lifestyle and Total psychopathic traits and Sexual Relationships, and a positive association between Interpersonal psychopathic traits and Admiration. Both studies also found a negative association between Affective psychopathic traits and Prosocial Interactions and Interpersonal psychopathic traits and Passivity, although Study 1 found these associations with all psychopathic traits. In addition, Lifestyle psychopathic traits were positively associated with Sociability in Study 1, but not Study 2.

In the social reward experimental task, a novel symbol of social reward was used: the ‘Like’ thumbs-up symbol from the social networking site Facebook. RTs to both the Like and pound symbol were faster with each incremental reward probability level. There were no significant differences between mean RTs in the monetary and social reward tasks. This suggests that the Like symbol was serving as a reward stimulus in a manner similar to monetary reward, and so it may have value in future studies of social reward.

Interpersonal psychopathic traits were positively associated with the monetary-social RT difference score in both the 0.5 and 1 probability level conditions. Specifically, as Interpersonal traits increased, RTs in the social task became faster relative to the monetary task. We interpret this in the context of the narcissism and manipulation associated with the Interpersonal factor [27]. Specifically, the Like symbol represents social admiration/approval, and so this symbol may have a higher subjective value for individuals who tend to trick and manipulate others.

## General Discussion

In the two studies reported here we explored associations between psychopathic traits and the value of different social rewards. The main finding from our studies was that individuals with high levels of psychopathic traits reported that they like behaving antisocially and dislike behaving prosocially towards others. Data from the experimental reward tasks suggested that individuals with high levels of Interpersonal psychopathic traits appeared to find social admiration/approval especially motivating relative to monetary reward. Together, these findings shed light on what might motivate the social behaviour characteristic of individuals with high levels of psychopathic traits.

The implication that individuals with high levels of psychopathic traits enjoy cruel behaviour is in line with findings from other studies (e.g. [15]). A careful consideration of sadism is important here, which is defined as the enjoyment of controlling, dominating, and/or causing suffering to others, and can refer to physical or psychological suffering [51]–[52]. There is some existing support that psychopathy and sadism are overlapping constructs [53]–[56], and the current study provides further support for this. However, it remains unclear exactly why individuals with high levels of psychopathic traits enjoy cruel behaviour. One possibility is that inflicting suffering on others may be pleasurable purely because of causing a person pain (physical or psychological). Alternatively, the enjoyment may stem from the power and control that comes with inflicting suffering, and it is this rather than the pain per se that has reward value. Further research should probe the exact nature of the Negative Social Potency reward that is associated with psychopathic traits, and this value in antisocial behaviour should be incorporated into explanations of why psychopaths behave so badly towards others.

In addition, the current study found a negative association between psychopathic traits and enjoyment of prosocial interactions (Study 1: all factors; Study 2: Affective factor only). This finding suggests that individuals with high levels of psychopathic traits do not just feel indifferent towards being kind and helpful, they find it unappealing. Psychopathic traits have previously been associated with an increased report of *public* prosocial behaviours but a decreased report of *anonymous* and *altruistic* prosocial behaviours [9]. This is consistent with the current findings as it appears that individuals with high levels of psychopathic traits do not experience an intrinsic reward from behaving prosocially towards others [9]. This contrasts with evidence from typical individuals, which shows that people behave prosocially at least in part because they experience inherent enjoyment from it (the ‘warm glow’ hypothesis of altruism; [57]–[58]). The absence of this enjoyment in individuals with high levels of psychopathic traits is an important avenue for further research as it likely contributions to their reduced levels of cooperative and prosocial behaviour (e.g. [8]).

It is important to note that not all significant associations between psychopathic traits and social reward in Study 1 were replicated in Study 2. For example, Prosocial Interactions were negatively associated with all psychopathic traits in Study 1, but only Affective psychopathic traits in Study 2. There are a number of possible explanations for these discrepancies. For example, the two samples were drawn from different populations and the sample in Study 1 completed the questionnaires online rather than in the presence of the experimenter. These factors or others could have contributed to the difference between the two samples. It is also important to note the effects of age and gender seen in the post-hoc analyses in Study 1. It will be valuable to study social reward and psychopathic traits further to fully understand the relationship between these two constructs and how this might be influenced by demographic characteristics. However, the fact that the association between all psychopathic traits and Negative Social Potency was found in both samples, despite their demographic differences, suggests this may be a particularly important aspect of social reward for individuals with high levels of psychopathic traits.

In Study 2, we also conducted two experimental reward tasks with the aim of further elucidating the relationship between psychopathic traits and social reward. There were no significant associations between psychopathic traits and RTs at any probability level of monetary or social rewards. However, a significant positive association was found between Interpersonal traits and monetary-social difference scores for the 0.5 and 1 probability levels. In other words, as Interpersonal traits increased, the RTs to possible reward became faster in the social task relative to the monetary task.

As there were no significant associations between Interpersonal traits and RTs to either monetary or social conditions, these difference score associations are not clearly explained by either slower RTs to monetary reward or faster RTs to social reward. Rather, it is the relative difference between these two rewards that appears important, suggesting that individuals with high levels of Interpersonal traits confer relatively stronger value for social than monetary reward. It is important to note the type of social reward that the Facebook Like symbol represents: approval or admiration of one’s actions or lifestyle. The Interpersonal dimension of psychopathy describes the manipulative use of others, for which winning other’s approval may be particular useful. This may partly explain the relative importance that individuals with high levels of these traits placed on this type of social reward. This speculation is supported by the self-report findings from both samples reported here that Interpersonal traits (but not other psychopathy factors) were positively associated with the enjoyment of Admiration.

We had hypothesised that psychopathic traits would be positively associated with RTs to monetary reward, but this was not supported. Previous studies have found that psychopathic traits are associated with increased neural responsiveness to monetary reward [19]–[20]. However, these associations were with neural responses, and have not been demonstrated behaviourally. Therefore, one explanation is that the association between psychopathic traits and hypersensitivity to monetary reward is only apparent at a neural level. In addition, both previous studies used a different measure of psychopathic traits (Psychopathic Personality Inventory; [59]) than the one used in the current study, which furthers limits the extent to which we can compare between studies. A hypersensitivity to financial gain may have important implications for behaviour, particularly in combination with other psychopathic characteristics such as impulsivity and a lack of empathy, so the relationship between psychopathic traits and monetary reward value is worthy of further clarification in future studies.

Some limitations to the present study should be noted. Firstly, the sample size of the second study is small and the experimental findings should be replicated with larger samples. The current analyses were also exploratory and correlational. It would be interesting to test more directional hypotheses using more sophisticated regression analyses in the future. For example, it would be interesting to explore whether Interpersonal psychopathic traits predict performance in a social reward task above and beyond the variance shared with other aspects of psychopathic personality. Secondly, difference scores can be difficult to interpret, and it is important to further probe the relative contribution of monetary and social reward value to fully understand the current association between Interpersonal psychopathic traits and the monetary-social difference scores in the experimental tasks. In addition, it would be helpful to collect data measuring the subjective value of the Like and pound symbols for each participant, to assess the impact of this on task performance. Finally, the current study used community samples, and so it will be important to explore if the same pattern of associations between social reward and psychopathic traits is present in clinical samples.

In summary, the current study presents evidence that individuals with high levels of psychopathic traits may have an inverted pattern of social reward: they devalue affiliative and prosocial interactions, and instead take pleasure in treating others cruelly. Our experimental evidence suggests that individuals with high levels of Interpersonal traits place particular value on gaining social approval, which we speculate may be due to their manipulative treatment of others and the usefulness of approval in this context. Research addressing social reward in psychopathy is in its infancy, and there are likely to be a host of different processes that contribute to the value of different types of social reward. An important future direction will be to extend the current findings by elucidating the mechanisms behind the ‘inverted’ social reward associated with psychopathic traits.

## Supporting Information

Table S1 Descriptives for Study 1 (N = 505).
(DOCX)

Table S2 Descriptives for SRQ and SRP scores in Study 2 (N = 110).
(DOCX)

Table S3 Means and SDs for RTs at each reward probability level in both social and monetary conditions.
(DOCX)

Table S4 Simple effects analysis of all probability levels in both social and monetary conditions.
(DOCX)

Table S5 Associations between SRP and SRQ in Study 1 (N = 505) controlling for age.
(DOCX)

Table S6 Associations between SRP and SRQ in Study 1 for males only (N = 270).
(DOCX)

Table S7 Associations between SRP and SRQ in Study 1 for females only (N = 235).
(DOCX)

Dataset S1 Dataset for Study 1 and Study 2.
(SAV)
