# Supplementary material for: Inverted Social Reward: Associations between Psychopathic Traits and Self-Report and Experimental Measures of Social Reward
Source: PLoS One. 2014 Aug 27;9(8):e106000. doi: 10.1371/journal.pone.0106000 (PMC4146585; doi:10.1371/journal.pone.0106000)
Supplement: Table S1 — Descriptives for Study 1 (N = 505). (DOCX) [file pone.0106000.s001.docx]

**Table S1.**

|  | Minimum | Maximum | Mean (SD) |
| --- | --- | --- | --- |
| *SRQ subscale* |  |  |  |
| Admiration | 1.00 | 7.00 | 5.09 (1.14) |
| Negative Social Potency | 1.00 | 6.00 | 2.04 (1.09) |
| Passivity | 1.00 | 7.00 | 3.13 (1.27) |
| Prosocial Interactions | 2.60 | 7.00 | 5.98 (0.85) |
| Sexual Relationships | 1.00 | 7.00 | 5.06 (1.53) |
| Sociability | 1.00 | 7.00 | 4.61 (1.39) |
| *SRP subscale* |  |  |  |
| Affective | 7.00 | 30.00 | 14.21 (5.10) |
| Interpersonal | 7.00 | 33.00 | 14.01 (5.59) |
| Lifestyle | 7.00 | 35.00 | 15.13 (5.31) |
| Antisocial | 7.00 | 31.00 | 10.60 (4.34) |
| *SRP Total* | 28.00 | 122.00 | 53.96 (17.61) |
