# Supplementary material for: Inverted Social Reward: Associations between Psychopathic Traits and Self-Report and Experimental Measures of Social Reward
Source: PLoS One. 2014 Aug 27;9(8):e106000. doi: 10.1371/journal.pone.0106000 (PMC4146585; doi:10.1371/journal.pone.0106000)
Supplement: Table S2 — Descriptives for SRQ and SRP scores in Study 2 (N = 110). (DOCX) [file pone.0106000.s002.docx]

**Table S2.**

|  | Minimum | Maximum | Mean (SD) |
| --- | --- | --- | --- |
| *SRQ subscale* |  |  |  |
| Admiration | 2.50 | 7.00 | 5.73 (0.85) |
| Negative Social Potency | 1.00 | 4.80 | 2.27 (0.88) |
| Passivity | 1.00 | 5.70 | 3.00 (1.03) |
| Prosocial Interactions | 4.80 | 7.00 | 6.12 (0.54) |
| Sexual Relationships | 1.00 | 7.00 | 5.22 (1.30) |
| Sociability | 3.00 | 7.00 | 5.44 (0.87) |
| *SRP subscale* |  |  |  |
| Affective | 7.00 | 27.00 | 14.37 (3.86) |
| Interpersonal | 7.00 | 31.00 | 16.27 (4.63) |
| Lifestyle | 7.00 | 27.00 | 17.10 (4.35) |
| Antisocial | 7.00 | 18.00 | 9.75 (2.88) |
| Total | 29.00 | 85.00 | 57.49 (12.32) |
