# Supplementary material for: Inverted Social Reward: Associations between Psychopathic Traits and Self-Report and Experimental Measures of Social Reward
Source: PLoS One. 2014 Aug 27;9(8):e106000. doi: 10.1371/journal.pone.0106000 (PMC4146585; doi:10.1371/journal.pone.0106000)
Supplement: Table S3 — Means and SDs for RTs at each reward probability level in both social and monetary conditions. (DOCX) [file pone.0106000.s003.docx]

**Table S3.**

|  | Probability | Mean (SD) |
| --- | --- | --- |
| Monetary | 0 | 306.30 (42.98) |
|  | 0.5 | 291.79 (39.86) |
|  | 1 | 286.22 (36.30) |
| Social | 0 | 307.78 (43.72) |
|  | 0.5 | 296.80 (42.05) |
|  | 1 | 291.12 (36.76) |
