# Supplementary material for: Inverted Social Reward: Associations between Psychopathic Traits and Self-Report and Experimental Measures of Social Reward
Source: PLoS One. 2014 Aug 27;9(8):e106000. doi: 10.1371/journal.pone.0106000 (PMC4146585; doi:10.1371/journal.pone.0106000)
Supplement: Table S4 — Simple effects analysis of all probability levels in both social and monetary conditions. (DOCX) [file pone.0106000.s004.docx]

**Table S4.**

|  | Probability (*A*) | Probability (*B*) | Mean *A* – Mean *B* difference (SE) |
| --- | --- | --- | --- |
| Monetary | 0 | 0.5 | 14.51** (2.52) |
|  |  | 1 | 20.08** (2.84) |
|  | 0.5 | 1 | 5.57* (2.43) |
| Social | 0 | 0.5 | 10.98** (2.64) |
|  |  | 1 | 16.66** (2.45) |
|  | 0.5 | 1 | 5.68* (2.40) |

**p<.001,*p<.05
