# Supplementary material for: Inverted Social Reward: Associations between Psychopathic Traits and Self-Report and Experimental Measures of Social Reward
Source: PLoS One. 2014 Aug 27;9(8):e106000. doi: 10.1371/journal.pone.0106000 (PMC4146585; doi:10.1371/journal.pone.0106000)
Supplement: Table S5 — Associations between SRP and SRQ in Study 1 (N = 505) controlling for age. (DOCX) [file pone.0106000.s005.docx]

**Table S5.**

|  | SRP-SF subscale | | | | SRP-SF Total^a^ |
| --- | --- | --- | --- | --- | --- |
|  | Affective^a^ | Interpersonal^a^ | Lifestyle^a^ | Antisocial^b^ |  |
| *SRQ subscale* |  |  |  |  |  |
| Admiration | -.03 | .06^^^ | .03 | -.10*^+^ | -.00 |
| Negative Social Potency | .61** | .63** | .47** | .58** | .69** |
| Passivity | .06 | .10* | .09^^^ | .10* | .12** |
| Prosocial Interactions | -.42** | -.39** | -.26** | -.45** | -.45** |
| Sexual Relationships | .12** | .12** | .32** | .04 | .17** |
| Sociability | -.04 | .04 | .12** | .05 | .05 |

^a^Pearson correlation, ^b^Spearman correlation

**p<.01,*p<.05

^^^Association loses significance when age is controlled, ^+^Association gains significance when age is controlled
