# Supplementary material for: Inverted Social Reward: Associations between Psychopathic Traits and Self-Report and Experimental Measures of Social Reward
Source: PLoS One. 2014 Aug 27;9(8):e106000. doi: 10.1371/journal.pone.0106000 (PMC4146585; doi:10.1371/journal.pone.0106000)
Supplement: Table S6 — Associations between SRP and SRQ in Study 1 for males only (N = 270). (DOCX) [file pone.0106000.s006.docx]

**Table S6.**

|  | SRP-SF subscale | | | | SRP-SF Total |
| --- | --- | --- | --- | --- | --- |
|  | Affective^a^ | Interpersonal^a^ | Lifestyle^a^ | Antisocial^b^ |  |
| *SRQ subscale* |  |  |  |  |  |
| Admiration | -.01 | .13* | .06 | -.03 | .05 |
| Negative Social Potency | .61** | .62** | .45** | .60** | .69** |
| Passivity | .12* | .11 | .09 | **.24**^+^** | .18** |
| Prosocial Interactions | -.34** | -.29** | -.18** | -.41** | -.36** |
| Sexual Relationships | **-.03^+^** | .08 | .28** | **-.14*^+^** | .07 |
| Sociability | .01 | **.14*^+^** | .21** | .14* | **.15*^+^** |

^a^Pearson correlation, ^b^Spearman correlation

**p<.01,*p<.05

^+^Correlation coefficient significantly different to that in female sample
