# Supplementary material for: Inverted Social Reward: Associations between Psychopathic Traits and Self-Report and Experimental Measures of Social Reward
Source: PLoS One. 2014 Aug 27;9(8):e106000. doi: 10.1371/journal.pone.0106000 (PMC4146585; doi:10.1371/journal.pone.0106000)
Supplement: Table S7 — Associations between SRP and SRQ in Study 1 for females only (N = 235). (DOCX) [file pone.0106000.s007.docx]

**Table S7.**

|  | SRP-SF subscale | | | | SRP-SF Total |
| --- | --- | --- | --- | --- | --- |
|  | Affective^a^ | Interpersonal^a^ | Lifestyle^a^ | Antisocial^b^ |  |
| *SRQ subscale* |  |  |  |  |  |
| Admiration | .04 | .07 | .07 | -.08 | .04 |
| Negative Social Potency | .59** | .63** | .48** | .53** | .66** |
| Passivity | .05 | .17* | .14* | **-.00^+^** | .13 |
| Prosocial Interactions | -.45** | -.44** | -.29** | -.42** | -.48** |
| Sexual Relationships | **.16*^+^** | .07 | .30** | **.12^+^** | .18** |
| Sociability | -.07 | **-.05^+^** | .04 | -.03 | **-.04^+^** |

^a^Pearson correlation, ^b^Spearman correlation

**p<.01,*p<.05

^+^Correlation coefficient significantly different to that in male sample
